# Health-related quality of life in overweight German children and adolescents: do treatment-seeking youth have lower quality of life levels? Comparison of a clinical sample with the general population using a multilevel model approach

**DOI:** 10.1186/1471-2458-13-561

**Published:** 2013-06-08

**Authors:** Emily Finne, Thomas Reinehr, Anke Schaefer, Katrin Winkel, Petra Kolip

**Affiliations:** 1 School of Public Health, Bielefeld University, Universitätsstraße 25, Bielefeld, D-33615, Germany; 2 Department of Pediatric Endocrinology, Diabetes and Nutrition Medicine, Vestische Youth Hospital, University of Witten/Herdecke, Dr.-F.-Steiner-Str. 5, Datteln, D-45711, Germany

**Keywords:** Health-related quality of life, Overweight, Obesity, Weight perception, Body image, Children and adolescents, Treatment-seeking status

## Abstract

**Background:**

Health-related quality of life (HRQoL) is reduced in obese children and adolescents, especially in clinical samples. However, little is known regarding the HRQoL of moderately overweight youth. Moreover, several studies have indicated perceived overweight as a critical factor associated with lower HRQoL. Our main objective was to compare HRQoL between treatment-seeking overweight youth and the general adolescent population, whilst separating the effects of treatment-seeking status and perceived weight from those of objective weight status.

**Methods:**

We compared the HRQoL of a clinical sample of overweight youth (N=137 patients, mean age±s.e.=11.24±0.15 years) with that of a representative population sample (N=6354, mean age=12.75±0.03 years). The population sample was subdivided into groups based on measured and perceived weight status. We used hierarchical linear models to compare HRQoL subscale scores (self- and parent-reported) between patients and population groups, adjusted for sociodemographic characteristics and taking into account clustering of the population sample.

**Results:**

The parent-reported HRQoL of the treatment sample was significantly lower than that of other overweight youth perceived as ‘too fat’ on two subscales: ‘self-esteem’ and ‘friends’ (effect sizes: d=0.31 and 0.34, respectively). On other subscales, patients scored lower than adolescents perceived as having a ‘proper weight’ by their parents. The patterns for self-reported HRQoL in adolescents were different: patients reported higher self-esteem than other overweight youth feeling ‘too fat’ (d=-0.39). Female patients also reported higher physical well-being (d=-0.48), whereas males scored lowest among all compared groups (d=0.42-0.95). Patients did not differ from other overweight youth who felt ‘too fat’ with respect to other HRQoL dimensions. In general, lower HRQoL was primarily associated with a perceived, rather than actual, overweight status.

**Conclusions:**

The treatment-seeking status of overweight youth was notably associated with low social well-being, which may therefore be the main motive for seeking treatment. Other HRQoL domains were not consistently reduced in treatment-seekers. Our results further indicate that perceived overweight rather than actual overweight impacts HRQoL in youth with a modest excess weight. These results have implications for interventions in overweight youth and in individuals who are dissatisfied with their weight.

**Trial registration:**

‘Obeldicks light’ is registered at clinicaltrials.gov (NCT00422916).

## Background

Obesity and overweight pose an increasing health problem in most industrialized countries [1-3]. The recent German Health Interview and Examination Survey for Children and Adolescents (KiGGS) reveals that 8.7% of children and adolescents aged 3**-**17 years meet the national definition of being overweight, and an additional 6.3% are classified as obese [4]. Compared with reference populations from the 1980s and 1990s, the prevalence of pediatric overweight and obesity in Germany has increased by approximately 50% [4].

The psychosocial consequences of excess weight in childhood are of specific concern, as being overweight is strongly stigmatized in Western society [5,6]. Because limitations in well-being may not be associated with clinical diagnoses but do impact many domains of everyday life, a quality of life approach seems especially suitable for describing overweight youth in terms of psychosocial functioning and well-being [7]. Health-related quality of life (HRQoL) relates to the self-perceived health of a person and consists of ratings of well-being and functionality in important life areas, including physical well-being/functioning, bodily symptoms, emotional well-being, self-esteem, social functioning, and family relations [8,9]. While disease-specific measures focus on impairments due to a specific health condition, generic HRQoL instruments enable comparisons between different health conditions or with healthy subjects [10].

Impairments in different dimensions of HRQoL are often associated with excess weight in adolescence [10-13], and the magnitude of excess weight is negatively associated with HRQoL [10,14,15], with HRQoL values of overweight youth lying between those of normal-weight and obese adolescents.

HRQoL seems especially impaired in overweight or obese youth seeking treatment, and this may be one motive for treatment-seeking. Some studies show marked differences in HRQoL between treatment-seeking and population-based samples of obese youth [16-18]. However, most of the available results were obtained from extremely obese individuals. In addition, some studies mixed up weight status with treatment-seeking status by comparing clinical samples of obese individuals with normal-weight controls; therefore, it remains unclear to what extent impairments are related to the weight of clinical samples instead of treatment-seeking status. Furthermore, a reduction in HRQoL of clinical samples seems directly related to the intensity of intended treatment, as well as BMI [14,19-21], so that results reported for obese clinical samples may not generalize to overweight patients presenting for outpatient training. Moderately overweight youth are a largely ignored group in the treatment literature. From a public health perspective, they should receive more attention, since it seems preferable to prevent further weight gain in this (large) group than to treat more extreme excess weight that has already entailed adverse health effects. To facilitate adaptation interventions to the specific needs of this target group, more needs to be known about specific aspects of well-being that may be impaired.

A factor that was also rarely accounted for when comparing overweight treatment samples to the general population is perceived weight status. Based on KiGGS data, Kurth and Ellert [22] reported that HRQoL predominantly varied with the subjectively perceived, rather than objectively measured, weight status. However, this association was only investigated in obese adolescents. International studies also revealed that associations between being overweight or obese with mental health or psychological well-being were explained by perceived weight or body dissatisfaction in adolescents [23-25]. This finding points to perceived weight as a critical factor to consider when investigating associations between weight and well-being in children and adolescents.

In the present study, we focused on moderately overweight but not obese children and adolescents and analyzed whether treatment-seeking overweight youth exhibit impairments in HRQoL similar to those found in clinical samples of obese children and adolescents. Since in children and adolescents treatment decisions may in large parts depend on parents’ perceptions, both self- and proxy-reported HRQoL were examined. While self-reported HRQoL may be more valid in terms of subjective well-being, parent-perceived HRQoL may impact more on treatment decisions.

Our main objectives were a) to describe the self-reported and parent-reported HRQoL of treatment-seeking moderate overweight youth, b) to compare it with the HRQoL of same-aged youth from the general population differentiated by objective as well as perceived weight status, and c) to explore possible sociodemographic moderators of HRQoL differences due to treatment status.

We expected a lower HRQoL in patients compared to overweight youth of the general population especially in terms of proxy-reports. We also supposed that patients would be more similar to other youth perceived as too fat than to those perceived as having a proper weight. Since knowledge on moderately overweight children and adolescents is sparse, we did not formulate hypotheses concerning specific aspects of HRQoL.

## Methods

### Samples and procedures

A clinical sample of overweight youth participating in the ‘Obeldicks light’ intervention study was compared with a population sample of similar age from the nationally representative German Health Interview and Examination Survey (KiGGS) [26]. Comparisons between these samples were possible because identical instruments were used to collect the data of interest.

#### Treatment sample

We recruited our sample from a study that aimed to evaluate the training program ‘Obeldicks light’ in terms of weight reduction, nutrition, physical activity, and HRQoL. ‘Obeldicks light’ is a six-month outpatient training program for overweight but not obese children and adolescents offered at two clinics in North-Rhine Westphalia in the West of Germany. The study was designed as a randomized controlled trial (RCT) with a waiting list control group to assess the effects of the training program on weight reduction and secondary outcomes. Participants were recruited mainly by local media and pediatricians. Details of the intervention and RCT are described elsewhere [27,28].

In the present analyses, we used baseline data from all eligible boys and girls who enrolled in the treatment program between January 2007 and August 2010 (hereafter also referred to as ‘patients’: N=137; including N=66 participants of the RCT, N=19 participants of a pilot study, and N=52 enrolled after the recruitment period for the RCT). To be included in the study, children had to be between 8 and 16 years old, overweight, apparently healthy and not be taking any medication. Overweight was defined as a BMI ≥90th percentile and ≤97th percentile, according to German percentiles [29]. All reported baseline measures were obtained at the study locations after enrollment in the study and before randomization and treatment administration.

The local ethics committee of the University of Bremen approved the study. Written informed consent was obtained from all subjects and their parents before the beginning of the study.

#### KiGGS

A total of 17,641 boys and girls aged 0-17 years and their parents participated in the survey from May 2003 to May 2006. The aims and methodology of the survey are described in detail elsewhere [30]. In brief, a stratified multistage probability sample representative of this age group in Germany was obtained: First, a stratified sample of 167 German communities was drawn and second, invited participants were randomly sampled from local population registries of these communities. The survey was announced in local media, and parents of the selected children were invited to participate by letters. The overall response rate was 66.6%. In the 167 local study centers (sample points), boys and girls and their parents responded to different versions of a questionnaire, and children were physically examined by a study team led by a physician. The study was approved by the Charité/Universitätsmedizin Berlin ethics committee and the Federal Office for the Protection of Data.

**Figure 1:**
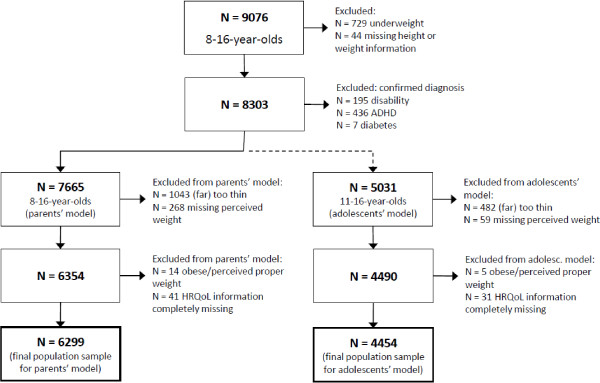
**Study flow chart for the selection of the analyzed population sample from the KiGGS study. **Note: ADHD: attention deficit hyperactivity disorder.

The present analyses are restricted to the subsample of 8-16-year-olds (N=9076) because this age range was covered by both studies included in our comparisons. Of this sample population, underweight individuals and those with missing or imprecise weight or height measurements were excluded. Subjects with confirmed disabilities, attention deficit hyperactivity disorder, or diabetes were also excluded because these factors are associated with HRQoL and affected children would not have been enrolled in the ‘Obeldicks light’ program. For comparisons of HRQoL scores, only individuals with perceived weights of ‘(far) too fat’ or ‘proper weight’ were included in the analyses. Figure 1 illustrates the selection process of the population sample from the KiGGS survey.

### Measures

#### Sociodemographic measures

Socioeconomic status (SES) and ethnic background in both samples were assessed by identical parent questionnaires. SES was based on parents’ education, occupation, and household income, with higher values indicating a higher status [31,32]. Regarding ethnicity, children whose parents were both immigrants or of non-German citizenship and those who were immigrants themselves and had at least one parent of non-German descent were classified as immigrants [33] and compared with children of German descent. Parents were asked who completed the questionnaire. Because the mother completed the questionnaire in most cases, a binary variable ‘mother vs. other caregiver’, was created. Other parameters considered were age and gender.

#### Weight-related measures

In both samples, standing height was measured to the nearest centimeter using a rigid stadiometer. Weight was measured, with the subjects in their underwear, to the nearest 0.1 kg using a calibrated balance scale. Body mass index (kg/m^2^) reference data for German children were used [30] to classify children as normal-weight (10th-<90th percentile), overweight (90th-97th percentile) or obese (>97th percentile). Children with a BMI <10th percentile (‘underweight’) were excluded from the analyses.

Because the BMI of the parents may impact their HRQoL judgments [34], the BMI of the proxy-rater was also considered when assessing the parent-reported HRQoL. The BMI values of the mother and father were calculated based on their self-reported weight and height. When the questionnaire was filled out by both parents, the mean BMI of the mother and father was used.

To assess perceived weight status in the KiGGS study, parents and adolescents (≥ 11 years of age) were asked whether they thought of the child as ‘far too thin’, ‘a bit too thin’, ‘proper weight’, ‘a bit too fat’, or ‘far too fat’. Because very few overweight or obese children were rated as too thin, we limited our analyses to the categories of ‘proper weight’ and ‘(far) too fat’ (see Figure 1). The extreme categories were substantially less common (‘far too fat’ indicated by 4.3% of parents and 8.7% of adolescents, respectively). Because of resulting small group sizes, the categories ‘a bit too fat’ and ‘far too fat’ were combined. In the Obeldicks light study, parents rated their child’s weight in an analogous manner.

#### Health-related quality of life (HRQoL)

HRQoL was measured by age-specific self-report and parent proxy versions of the revised German KINDL-R questionnaire [35,36]. The KINDL is a generic HRQoL measure that distinguishes six dimensions of HRQoL with reference to the past week: physical (e.g., ‘I felt ill’) and emotional (e.g., ‘I had fun and laughed a lot’) well-being, self-esteem (e.g., ‘I was proud of myself’), family (e.g., ‘I got along well with my parents’), friends (e.g., ‘I got along well with my friends’), and school (e.g., ‘Doing the schoolwork was easy’). Each dimension is measured by four items and transformed to a score in the range of 0 (lowest HRQoL) to 100 (highest HRQoL). HRQoL was proxy-rated by parents in both studies. In the KiGGS study, self-report measures were only available for adolescents 11 years old and older. Self-reported values were therefore only compared between adolescents aged 11–16 years.

KINDL-R showed acceptable reliability and validity in different applications, including the KiGGS study [15,37,38]. For the treatment sample, internal consistencies of the subscales varied from α=0.42-0.76, with the lowest reliability found for the friends subscales, and values of α<0.70 were obtained for the self-reported and proxy-reported emotional well-being and school subscales, as well as for the parent-reported self-esteem and self-reported physical well-being subscales.

### Statistical analyses

#### Data screening and missing values

The percentage of missing values was well below 5% for each analyzed predictor in the selected sample. No cases had missing age information. Missing values for SES and parental BMI were imputed by the MVA regression method in SPSS 20.0 (IBM Corporation, Somers, NY, USA), using all potential variables of the final analyses as predictors of the imputed values.

Completed data were screened for distributional assumptions. In general, HRQoL values deviated from a normal distribution. All analyses were therefore rechecked after performing a normalizing transformation. Because the results derived from the transformed values were very similar to those from raw values and the inferences were the same, for ease of interpretability, only the results of the untransformed values are reported.

#### Descriptive statistics

Descriptive statistics for the sociodemographic measures and weight status of both samples were compared using independent t-tests and chi-square tests. The means and standard errors of HRQoL subscale scores were computed for groups distinguished by objective and perceived weight status. All descriptive statistics were computed using the ComplexSamples procedures of the SPSS software, taking into account the clustering and individual case weighting of the survey.

#### Main analyses: Group comparisons of patients with population groups

To compare HRQoL scores of the treatment sample with those of different groups from the KiGGS sample, three-level hierarchical linear regression models were analyzed with the software HLM 6.08 (Scientific Software International, Skokie, IL, USA) with HRQoL subscale scores (level 1) nested in subjects (level 2) nested in sample points (level 3). In the treatment sample each person was considered as one sample point. This procedure allowed us to maintain sampling information while simultaneously analyzing the groups in a comparable manner. All predictors were measured at the subject level (level 2). One model was computed to compare parent-proxy-reported HRQoL scores between the entire sample population, and one model was computed to compare the self-reported scores between the 11-16-year-olds.

The objective and perceived weight statuses were combined to form the comparison groups within the population sample. For this purpose, in the parents’ model the weight status as perceived by parents was used, and the self-reported perceived weight was used in the children’s model. Because there were very few obese children perceived as having a ‘proper weight’, this group (N=14 in the parents’ model and N=5 in the children’s model) was excluded (see Figure 1).

The ‘group’ variable comparing patients with objective/subjective weight groups of the general population was recoded into five dummy variables used to compare each of the population groups with the treatment sample (as the reference group).

Since previous studies have used either normal-weight or overweight comparisons groups without considering perceived weight, for commensurable results additional comparisons were performed to compare patients with a) overweight and normal-weight youth from the general population (perceived ‘proper weight’ and ‘too fat’ combined) and b) youth from the general population perceived as ‘too fat’ (normal-weight, overweight, and obese combined).

Included as covariates were gender, age, immigration status, SES, and BMI z-score. In the parent model, the effects of the reporting person (i.e., the mother vs. another caregiver) and the BMI of the proxy-rater were also analyzed^a^.

HRQoL intercepts were allowed to vary between sample points, while group and covariate effects were fixed to be the same for all sample points. The error variance at level 1 was fixed to allow for estimation. To adjust KiGGS data for deviations from representativeness regarding age, gender, region, and nationality, cases were weighted at the subject level. Robust standard errors were obtained for all model parameters.

Effect sizes (d) for the comparisons with the treatment sample were computed as the model-predicted mean difference divided by the person-level standard deviation. The results were classified as small (0.20), medium (0.50) or large (0.80).

## Results

### Descriptive statistics

Table 1 describes both samples in terms of sociodemographic and anthropometric measures, as well as perceived weight status. There were no differences between the samples with regard to socioeconomic status or proportion of mothers as proxy-raters. However, the proportions of females and adolescents were higher in the treatment sample than in the population sample, while the proportion of immigrants was significantly lower. The treatment sample was also significantly younger on average, and their parents self-reported significantly higher BMIs. As expected, patients had a higher BMI z-score than the general population, and all were overweight. With nearly all of the patients’ weight rated as ‘(far) too fat’, the perceived weight also significantly differed between the samples. Because there was almost no variation in this group perceived weight was not differentiated within the treatment sample.

**Table 1:** Description of the sample - KiGGS vs. treatment sample groups

	**KiGGS sample (8–16 years)**	**Obeldicks light treatment sample**	**p-value**
N	6354 (unweighted)	137	
% female	53.0	62.8	<0.01
% adolescents (≥ 11 year olds)	30.5	52.6	<0.001
Age: M (s.e.)	12.75 (0.03)	11.24 (0.15)	<0.001
% HRQoL proxy- report by mother	83.7	81.4	n.s.
Proxy BMI: M (s.e.)	25.28 (0.07)	27.47 (0.32)	<0.001
SES score: M (s.e.)	11.60 (0.10)	11.12 (0.34)	n.s.
BMI z-score	0.53 (0.01)	1.62 (0.01)	<0.001
% immigrants	15.6	9.5	<0.05
**Weight categories:**			<0.001
% normal-weight	79.1	-	
% overweight	11.9	100	
% obese	9.0	-	
**Perceived weight status: parent-reported (N=6354 KiGGS, N=137 Obeldicks light)**			
% proper weight	70.8	0.7	<0.001
% a bit too fat	24.7	78.8	
% far too fat	4.5	20.5	
**Perceived weight status: self-reported (N=4490 KiGGS)**			
% proper weight	47.3	-	
% a bit too fat	44.0	-	
% far too fat	8.7	-	

Note: p-values are based on independent samples t-tests for continuous variables and χ2 tests for categorical variables. n.s. = not significant (p>0.05); s.e. = standard error.

The mean HRQoL scores of girls and boys in the compared groups and the entire KiGGS sample are displayed in Table 2.

**Table 2:** Descriptive statistics for the HRQoL subscale scores in the compared objective/perceived weight groups in the population and the treatment sample (mean; standard error)

		**Population mean**^**†**^	**Normal-weight/ proper weight**	**Overweight/ proper weight**	**Normal-weight/ too fat**	**Overweight/ too fat**	**Obese/ too fat**	**Treatment sample**
**Parent proxy-reported HRQoL (8–16 years old)**								
		N = 9076 ^‡^	N=4294	N=149	N=688	N=660	N=549	N=137
Physical well-being	boys	77.84; 0.29	80.86; 0.39	80.05; 1.95	75.25; 1.22	76.70; 1.03	74.17; 1.22	75.80; 2.40
	girls	74.50; 0.33	76.30; 0.43	78.60; 1.93	72.25; 1.13	71.35; 1.05	68.53; 1.17	74.08; 2.01
Emotional well-being	boys	80.09; 0.22	81.76; 0.27	82.18; 1.54	78.79; 0.88	79.21; 0.98	80.02; 0.83	76.04; 2.11
	girls	80.03; 0.25	81.38; 0.31	84.12; 1.63	78.07; 0.97	78.06; 0.82	77.10; 1.04	78.04; 1.55
Self-esteem	boys	68.18; 0.26	69.73; 0.36	72.11; 1.41	65.81; 0.97	67.21; 1.04	68.40; 0.95	62.87; 2.23
	girls	68.29; 0.25	69.60; 0.33	71.20; 1.93	65.63; 0.88	66.21; 1.04	65.93; 1.07	63.23; 1.77
Family	boys	76.99; 0.25	78.25; 0.36	78.12; 1.82	75.18; 0.93	77.38; 0.88	79.35; 0.95	77.04; 2.05
	girls	77.69; 0.25	78.77; 0.34	77.63; 2.70	75.46; 0.91	76.75; 0.93	76.72; 1.16	75.58; 1.61
Friends	boys	77.56; 0.25	79.57; 0.29	78.80; 1.91	76.29; 1.00	77.18; 0.90	76.52; 1.03	71.41; 2.32
	girls	77.15; 0.24	78.39; 0.30	81.42; 1.71	75.50; 0.78	74.37; 0.96	73.35; 1.36	71.06; 1.30
School	boys	73.78; 0.27	75.73; 0.37	74.65; 1.52	71.94; 1.15	73.95; 1.15	73.17; 1.10	72.07; 2.35
	girls	75.77; 0.31	76.76; 0.39	73.60; 2.12	75.85; 1.07	76.24; 0.90	71.54; 1.16	77.22; 1.79
**Children’s self-reported HRQoL (11–16 years old)**								
		N = 5954	N=2063	N=62	N=1485	N=494	N=381	N=65
Physical well-being	boys	74.50; 0.32	77.16; 0.44	79.83; 2.38	73.38; 0.74	72.99; 1.09	71.50; 1.31	66.09; 3.76
	girls	67.97; 0.40	72.10; 0.62	72.26; 3.56	65.22; 0.67	64.14; 1.21	65.36; 1.28	74.01; 2.43
Emotional well-being	boys	82.27; 0.25	83.52; 0.39	86.21; 1.61	81.67; 0.55	80.82; 0.93	82.88; 0.89	79.28; 3.27
	girls	80.75; 0.32	83.26; 0.45	85.61; 2.69	79.21; 0.57	80.18; 0.99	78.43; 1.36	82.73; 1.77
Self-esteem	boys	60.11; 0.38	61.81; 0.59	63.02; 2.81	58.76; 0.86	57.96; 1.29	56.56; 1.43	60.42; 3.85
	girls	55.85; 0.37	60.27; 0.60	65.90; 4.95	53.20; 0.67	52.71; 1.38	52.36; 1.84	61.57; 2.25
Family	boys	82.54; 0.28	83.77; 0.47	89.31; 2.39	81.41; 0.67	82.45; 1.17	82.22; 1.19	81.25; 2.69
	girls	81.58; 0.34	84.74; 0.58	79.74; 3.48	78.66; 0.61	79.58; 1.12	82.93; 1.13	78.73; 2.84
Friends	boys	78.78; 0.32	80.63; 0.44	80.80; 2.18	78.82; 0.71	77.13; 1.14	74.79; 1.28	71.99; 3.30
	girls	76.83; 0.32	78.70; 0.49	77.68; 2.70	75.92; 0.54	75.65; 1.11	74.71; 1.63	75.00; 2.21
School	boys	66.57; 0.38	69.29; 0.56	72.36; 3.04	64.66; 0.81	64.62; 1.25	63.84; 1.43	66.20; 3.48
	girls	66.33; 0.37	70.57; 0.64	74.61; 5.87	63.04; 0.59	64.05; 1.38	62.89; 1.88	68.26; 2.87

Note: ^†^ Based on the KiGGS sample before the exclusion of underweight and disabled boys and girls (8–16 years old for the analysis of parent-reported values and 11–16 years old for the analysis of self-reported values).

‡ N denotes group numbers; individual Ns may differ for different HRQoL scores due to missing values).

Worth remarking, HRQoL differences between overweight and normal-weight children within the perceived weight categories seemed rather small. Furthermore, the differences between obese and overweight youth who were rated as ‘too fat’ were generally small in relation to the differences between perceived weight categories. These patterns were similar between boys and girls.

### Hierarchical model of parent-reported HRQoL

Table 3 shows the results of the final three-level model of parent-reported HRQoL scores. The main results are the fixed effects for the group comparison of patients with objective/subjective weight groups from the population sample. The detailed interpretation of the results of Tables 3 and 4 is described in Additional file 1.

**Table 3:** Results of the three-level hierarchical linear regression model comparing parent proxy-reported HRQoL scores between patients and objective/subjective weight groups from the general population

**FIXED EFFECTS**	**Coefficient**	**Robust s.e.**	**t-ratio**	**Effect size d**^**‡**^	**FIXED EFFECTS**	**Coefficient**	**Robust s.e.**	**t-ratio**	**Effect size d**^**‡**^
**Physical well-being:**					**Family:**				
Intercept	71.494	1.550	46.121***		Intercept	74.971	1.257	59.624***	
Sex (male)	4.156	0.420	9.895***	0.26	Sex (male)	-0.209	0.413	-0.506	-0.01
Proxy not mother	2.348	0.655	3.586***	0.15	Proxy not mother	1.523	0.602	2.529*	0.11
Immigrant	-0.629	0.705	-0.893	-0.04	Immigrant	3.100	0.652	4.757***	0.22
Age†	-1.085	0.089	-12.206***	-0.20^§^	Age†	-0.588	0.075	-7.834***	-0.13^§^
SES score†	0.250	0.058	4.278***	0.09^$^	SES score†	0.111	0.049	2.276*	0.05^$^
***Group effects ****(ref.: Obeldicks light)*	Multivariate hypothesis χ^2 ^with robust s.e.: χ^2^_(df=5) _=114.521***				***Group effects ****(ref.: Obeldicks light)*	Multivariate hypothesis χ^2 ^with robust s.e.: χ^2^_(df=5) _=28.512***			
Normal/proper	4.961	1.569	3.162**	0.31	Normal/proper	3.203	1.269	2.525*	0.23
Normal/too fat	0.111	1.755	0.063	0.01	Normal/too fat	-0.118	1.418	-0.083	0.01
Overweight/proper	5.925	2.088	2.837**	0.37	Overweight/proper	2.644	2.026	1.305	0.19
Overweight/too fat	0.553	1.684	0.328	0.03	Overweight/too fat	1.376	1.407	0.978	0.10
Obese/too fat	-1.454	1.741	-0.835	-0.09	Obese/too fat	2.694	1.465	1.838	0.19
**Emotional well-being:**					**Friends:**				
Intercept	76.469	1.260	60.679***		Intercept	70.093	1.198	58.488***	
Sex (male)	0.534	0.357	1.496	0.04	Sex (male)	1.349	0.357	3.778***	0.10
Proxy not mother	0.489	0.478	1.023	0.04	Proxy not mother	1.129	0.526	2.145*	0.09
Immigrant	0.222	0.574	0.387	0.02	Immigrant	0.631	0.665	0.948	0.05
Age†	0.490	-0.065	-7.500***	0.12^§^	Age†	-0.216	0.072	-3.022**	-0.05^§^
SES score†	0.131	0.040	3.291***	0.06^$^	SES score†	-0.130	0.045	-2.902**	-0.06^$^
***Group effects****(ref.: Obeldicks light)*	Multivariate hypothesis χ^2 ^with robust s.e.: χ^2^_(df=5) _=60.930***				***Group effects****(ref.: Obeldicks light)*	Multivariate hypothesis χ^2 ^with robust s.e.: χ^2^_(df=5) _=101.758***			
Normal/proper	4.853	1.269	3.824***	0.39	Normal/proper	8.104	1.214	6.674***	0.61
Normal/too fat	1.549	1.392	1.113	0.12	Normal/too fat	5.045	1.327	3.800***	0.38
Overweight/proper	6.377	1.719	3.710***	0.51	Overweight/proper	8.580	1.843	4.655***	0.65
Overweight/too fat	1.957	1.403	1.395	0.16	Overweight/too fat	4.505	1.379	3.266***	0.34
Obese/too fat	2.224	1.427	1.558	0.18	Obese/too fat	3.650	1.487	2.454*	0.28
**Self-esteem:**					**School:**				
Intercept	62.218	1.395	44.609***		Intercept	74.993	1.354	55.384***	
Sex (male)	-0.195	0.432	-0.452	0.01	Sex (male)	-2.258	0.471	-4.796***	-0.16
Proxy not mother	1.265	0.934	1.354	0.09	Proxy not mother	-1.927	0.773	-2.492*	-0.13
Immigrant	-0.412	0.719	-0.573	0.03	Immigrant	-6.772	0.737	-9.185***	-0.47
Age†	-0.520	0.073	-7.149***	-0.11^§^	Age†	-1.906	0.083	-22.888***	-0.39^§^
SES score†	0.160	0.048	3.335***	0.07^$^	SES score†	0.448	0.045	9.859***	0.18^$^
Interaction child gender by proxy-rater	2.516	1.066	2.360*	0.18	Interaction child gender by proxy-rater	3.293	0.968	3.402***	0.23
***Group effects****(ref.: Obeldicks light)*	Multivariate hypothesis χ^2 ^with robust s.e.: χ^2^_(df=5) _=75.984***				***Group effects****(ref.: Obeldicks light)*	Multivariate hypothesis χ^2 ^with robust s.e.: χ^2^_(df=5) _=18.589**			
Normal/proper	7.317	1.400	5.225***	0.51	Normal/proper	3.425	1.365	2.510*	0.24
Normal/too fat	3.341	1.512	2.210*	0.23	Normal/too fat	1.201	1.543	0.778	0.08
Overweight/proper	9.319	1.832	5.087***	0.65	Overweight/proper	2.960	1.834	1.614	0.20
Overweight/too fat	4.390	1.559	2.816**	0.31	Overweight/too fat	2.725	1.514	1.800	0.19
Obese/too fat	5.203	1.552	3.353***	0.36	Obese/too fat	1.270	1.523	0.834	0.09
**RANDOM EFFECTS**	**Standard Deviation**	**Variance component**	**Chi-square**		**RANDOM EFFECTS**	**Standard Deviation**	**Variance component**	**Chi-square**	
**Level 2:**					**Level 3:**				
Physical well-being	16.059	257.895	1459059.004***		Physical well-being	2.017	4.068	413.866***	
Emotional well-being	12.538	157.210	884875.385***		Emotional well-being	1.136	1.291	381.487***	
Self-esteem	14.322	205.109	1124020.870***		Self-esteem	1.313	1.723	374.844***	
Family	14.074	198.066	1119831.448***		Family	1.537	2.363	366.903**	
Friends	13.216	174.672	988605.745***		Friends	1.108	1.227	340.837*	
School	14.354	206.030	1185310.926***		School	1.342	1.800	351.198*	

Note: * p≤ 0.05, ** p≤ 0.01, *** p≤ 0.001.

†Grand-mean centered.

‡ Effect size (d) computed as the model-estimated mean difference divided by the level 2-standard deviation of the subscale score.

§ Effect size for the mean difference of a 3-year increase in age.

$ Effect size for the mean difference of a 6-point increase in SES score (on a scale ranging from 3 to 21; corresponding to the difference between low and medium or medium and high SES, respectively).

s.e. = Standard error.

ref. = Reference group.

N=38,032 measurements nested in N=6436 individuals and N=304 sample points.

Model statistics: Deviance = 301,365.943; number of estimated parameters = 110.

**Table 4:** Results of the three-level hierarchical linear regression model comparing self-reported HRQoL scores in adolescents between patients and objective/subjective weight groups from the general population

**FIXED EFFECTS**	**Coefficient**	**Robust s.e.**	**t-ratio**	**Effect size d**^**‡**^		**FIXED EFFECTS**	**Coefficient**	**Robust s.e.**	**t-ratio**	**Effect size d**^**‡**^
**Physical well-being:**						**Family:**				
Intercept	71.729	2.364	30.345***			Intercept	78.492	1.988	39.490***	
Sex (male)	-6.502	3.859	-1.685	-0.39		Sex (male)	0.701	0.523	1.341	0.05
Immigrant	-1.584	0.148	-10.690***	-0.10		Immigrant	0.261	0.719	0.363	0.02
Age†	-0.002	0.060	-0.032	0.00^§^		Age†	-0.908	0.151	-6.009***	-0.17^§^
SES score†	-0.390	0.672	-0.581	-0.15^$^		SES score†	0.065	0.058	1.114	0.02^$^
***Group effects****(ref.: Obeldicks light)*	Multivariate hypothesis χ^2 ^with robust s.e.: χ^2^_(df=5) _=72.265***			♀	♂	***Group effects****(ref.: Obeldicks light)*	Multivariate hypothesis χ^2 ^with robust s.e.: χ^2^_(df=5) _=51.845***			
Normal/proper	0.045	2.423	0.019	0.00	0.81	Normal/proper	5.306	2.003	2.649**	0.35
Normal/too fat	-5.372	2.455	-2.188*	-0.35	0.53	Normal/too fat	1.176	2.030	0.579	0.08
Overweight/proper	-0.140	3.850	-0.036	-0.01	0.95	Overweight/proper	6.427	2.763	2.326*	0.42
Overweight/too fat	-7.303	2.686	-2.719**	-0.48	0.50	Overweight/too fat	2.118	2.099	1.009	0.14
Obese/too fat	-5.603	2.612	-2.145*	-0.37	0.42	Obese/too fat	4.133	2.175	1.900	0.27
***Interaction sex(male) × group***	Multivariate hypothesis χ^2 ^with robust s.e.: χ^2^_(df=5) _=14.804**					**Friends:**				
× normal/proper	12.407	3.908	3.175**			Intercept	71.686	1.913	37.466***	
× normal/too fat	13.488	3.943	3.421***			Sex (male)	1.976	0.499	3.960***	0.14
× overweight/proper	14.735	5.458	2.700**			Immigrant	-0.680	0.691	-0.985	-0.05
× overweight/too fat	14.959	4.209	3.554***			Age†	-1.196	0.142	-8.393***	-0.25^§^
× obese/too fat	12.095	4.120	2.936**			SES score†	-0.206	0.062	-3.335***	-0.08^§^
**Emotional well-being:**						***Group effects****(ref.: Obeldicks light)*	Multivariate hypothesis χ^2 ^with robust s.e.: χ^2^_(df=5) _=49.050***			
Intercept	80.064	1.728	46.335***			Normal/proper	7.263	1.944	3.735***	0.50
Sex(male)	1.257	0.434	2.898**	0.10		Normal/too fat	5.197	1.964	2.646**	0.36
Immigrant	-0.539	0.621	-0.868	-0.04		Overweight/proper	6.402	2.600	2.463*	0.44
Age†	-0.739	0.125	-5.914***	-0.18^§^		Overweight/too fat	3.669	2.075	1.768	0.25
SES score†	0.059	0.053	1.114	0.03^$^		Obese/too fat	2.309	2.228	1.036	0.16
***Group effects****(ref.: Obeldicks light)*	Multivariate hypothesis χ^2 ^with robust s.e.: χ^2^_(df=5) _=46.964***					**School:**				
Normal/proper	2.768	1.750	1.582	0.22		Intercept	66.218	2.155	30.729***	
Normal/too fat	-0.050	1.775	-0.028	0.00		Sex (male)	-0.212	0.512	-0.414	-0.01
Overweight/proper	5.221	2.261	2.309*	0.42		Immigrant	-5.532	0.834	-6.630***	-0.35
Overweight/too fat	-0.093	1.875	-0.050	-0.01		Age†	-2.003	0.165	-12.120***	-0.38^§^
Obese/too fat	0.390	1.925	0.202	0.03		SES score†	0.429	0.070	6.116***	0.16^$^
**Self-esteem:**						***Group effects****(ref.: Obeldicks light)*	Multivariate hypothesis χ^2 ^with robust s.e.: χ^2^_(df=5) _=95.841***			
Intercept	60.474	2.143	28.221***			Normal/proper	4.532	2.184	2.075*	0.28
Sex(male)	3.341	0.585	5.716***	0.19		Normal/too fat	-0.843	2.212	-0.381	-0.05
Immigrant	0.910	0.828	1.099	0.05		Overweight/proper	8.666	3.719	2.330*	0.54
Age†	1.022	0.173	5.907***	0.17^§^		Overweight/too fat	-0.229	2.323	-0.099	-0.01
SES score†	-0.014	0.074	-0.192	0.00^$^		Obese/too fat	-0.303	2.435	-0.124	-0.02
***Group effects****(ref.: Obeldicks light)*	Multivariate hypothesis χ^2 ^with robust s.e.: χ^2^_(df=5) _=83.703***									
Normal/proper	-1.405	2.181	-0.644	-0.08						
Normal/too fat	-7.093	2.204	-3.219**	-0.40						
Overweight/proper	1.572	3.706	0.424	0.09						
Overweight/too fat	-7.038	2.363	-2.979**	-0.39						
Obese/too fat	-8.235	2.428	-3.391***	-0.46						
**RANDOM EFFECTS**	**Standard Deviation**	**Variance component**	**Chi-square**			**RANDOM EFFECTS**	**Standard Deviation**	**Variance component**	**Chi-square**	
**Level 2:**						**Level 3:**				
Physical well-being	15.279	233.445	967288.672***			Physical well-being	1.630	2.657	291.914**	
Emotional well-being	12.367	152.952	638869.901***			Emotional well-being	1.010	1.020*	276.583*	
Self-esteem	17.884	319.822	1374249.579***			Self Esteem	0 (fixed)		ns	
Family	15.142	229.276	999529.880***			Family	0 (fixed)		ns	
Friends	14.610	213.444	929884.982***			Friends	0 (fixed)			
School	15.985	255.526	1064648.929***			School	1.993	3.972	317.574***	

Note: * p≤ 0.05, ** p≤ 0.01, *** p≤ 0.001, ns: not significant.

†Grand-mean centered.

‡ Effect size (d) computed as the model-estimated mean difference divided by the level 2-standard deviation of the subscale score.

§ Effect size for the mean difference of a 3-year increase in age.

$ Effect size for the mean difference of a 6-point increase in SES score (on a scale ranging from 3 to 21; corresponding to the difference between low and medium or medium and high SES, respectively).

s.e. = Standard error.

ref. = Reference group.

♀: girls, ♂: boys.

N=26,882 measurements nested in N=4519 individuals and N=232 sample points.

Model statistics: deviance = 218,590.150, number of estimated parameters = 92.

BMI z-score of the child was tested to control for differences between groups of the same weight category but showed no additional predictive value when added as covariate. Because the parental BMI had no significant effect on any of the HRQoL subscales, it was also not included in the final model. The only significant two-way interactions were found for child gender by proxy-rater on two subscales. With regard to age, non-linear effects were additionally explored but were found to be insignificant.

In general, mothers reported lower HRQoL scores for the subscales of physical well-being, family, and friends but higher scores for girls’ school-functioning than other proxies, and HRQoL scores varied with sociodemographic variables.

There were significant group effects on all HRQoL subscales. Various comparisons of the treatment sample with the general population demonstrated lower scores for the patients. The patients had a significantly lower physical and emotional well-being than normal-weight and overweight children rated ‘proper weight’ by parents, but they had similar values to those of normal-weight and other overweight children rated as ‘too fat’. With regard to the self-esteem and friends scales, patients scored significantly lower than all other groups, and the largest differences were observed between patients and children with a parent-perceived ‘proper weight’. Differences in the family and school scores were less pronounced between groups, and the only group from which the patients differed was the group with the highest HRQoL scores, namely the children with objective as well as perceived normal-weight.

The effect sizes for significant comparisons of the patients with other overweight youth rated ‘too fat’ were small (d=0.31-0.34), while small to moderate effect sizes (d=0.20-0.65) were found for comparisons with normal-weight youth rated as having a ‘proper weight’.

The deviance of the described final model was significantly lower than that of the null model (χ^2^_(df=62)_=1712.303, p<0.001) and of a model without the group predictors (χ^2^_(df=31)_=411.177, p<0.001).

**Figure 2:**
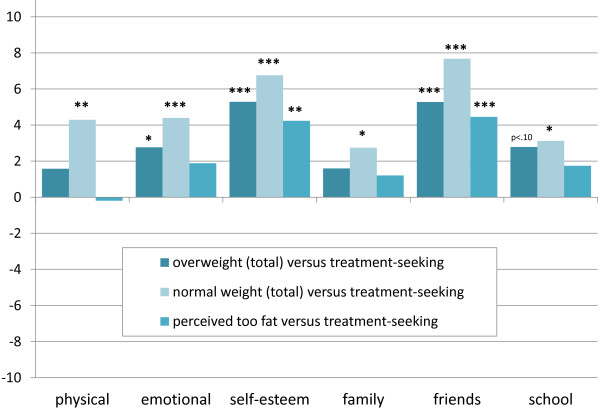
**Proxy-reported HRQoL of overweight, normal-weight youth, and youth perceived as ‘too fat’ in the general population compared with the treatment sample.** Note: 8–16 years old; N=6299 (KiGGS sample) and N=137 (Obeldicks light sample). *p≤0.05, ** p≤0.01., *** p≤0.001. Simple contrasts adjusted for sociodemographic differences and between-sample-point variation. Contrasts were performed within the described three-level hierarchical models but with other codes assigned to the compared groups.

When compared with overweight youth in the general population (independent of perceived weight), the patients exhibited significantly lower scores for parent-rated emotional well-being, self-esteem, and friends (see Figure 2). The parent-rated HRQoL of patients appeared to be impaired in all dimensions when compared with the HRQoL of normal-weight youth. However, compared with children and adolescents in the general population rated as ‘too fat’ (independent of objective weight), patients were only rated lower on the self-esteem and friends subscales.

### Hierarchical model of self-reported HRQoL in adolescents

The results of the hierarchical model of self-reported HRQoL in adolescents (11–16 years of age) are shown in Table 4. As in the parents’ model, BMI z-score showed no additional predictive value and was, therefore, not included in the final model.

There were significant group interaction effects for gender on physical well-being and for immigrant status on emotional well-being. All other interactions and non-linear age effects were not significant. Immigrants within the treatment sample self-reported markedly higher emotional well-being than did immigrants of all comparison groups, whereas no such group differences were observed in youth of German descent. However, this interaction was only apparent in comparisons with the treatment sample, and the number of immigrants in this sample (N=8) seems too small to draw general conclusions from this result. We therefore decided not to include this interaction effect in the final model.

For physical well-being, male patients reported significantly lower scores than all of the general population groups, while female patients had similar scores to those of females perceived as having a ‘proper weight’ and higher scores than other girls who perceived themselves as ‘too fat’ (see Table 2 for descriptive means; the same trend was found for emotional well-being but did not reach statistical significance). Emotional well-being scores were generally lower in the groups that felt ‘too fat’, with patients reporting similar values to those of other groups that felt ‘too fat’. The patient scores only significantly differed from those of the overweight/proper weight group.

With regard to school and familial well-being, patients differed mainly from the groups who felt they were having a ‘proper weight’, with patients reporting a lower HRQoL. Patients had significantly lower scores on the friends scale than all of the other groups, with the exception of the overweight/too fat (p<0.10) and obese/too fat (p>0.10) groups. Contrary to the parent-reported results, treatment-seeking youth had better self-esteem scores than did other youth in the population sample who felt ‘too fat’; in contrast, no difference was observed when comparing patients to groups who felt they were of the ‘proper weight’.

In general, the significant effects were of low to moderate size (d=0.22-0.54), although large effects were found for the comparison between male patients and males in the population sample who felt they were at the ‘proper weight’ (d=0.81 and 0.95, respectively).

The deviance of the described model was significantly lower than that of the null model (χ^2^_(df=56)_=1247.40, p<0.001) and of a model without group predictors (χ^2^_(df=35)_=316.43, p<0.001).

**Figure 3:**
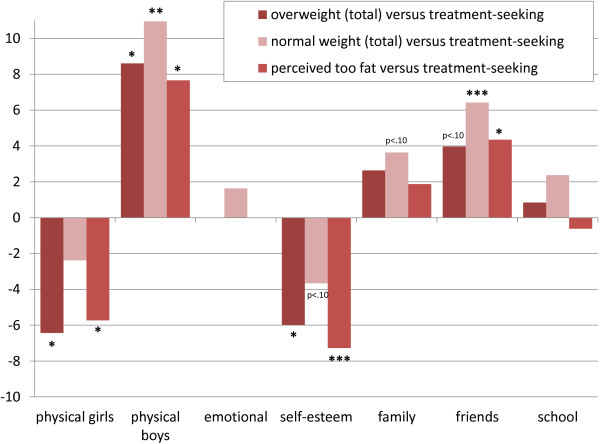
**Self-reported HRQoL of overweight, normal-weight youth, and youth perceived as ‘too fat’ in the general population compared with the treatment sample.** Note: 11–16 years old; N=4454 (KiGGS sample) and N=65 (Obeldicks light sample). *p≤0.05, ** p≤0.01, *** p≤0.001. Simple contrasts adjusted for sociodemographic differences and between-sample-point variation. Contrasts were performed within the described three-level hierarchical models but with other codes assigned to the compared groups.

Additional comparisons of patients with populations groups in terms of objective or subjective weight are illustrated in Figure 3. They revealed that treatment-seeking youth, contrary to their parents, rated their self-esteem significantly *higher* than overweight adolescents in the general population. In terms of physical well-being, treatment-seeking boys reported lower scores than normal-weight and overweight boys, while treatment-seeking girls reported significantly higher scores than other overweight girls. Male patients also rated their physical well-being significantly lower than other adolescents who felt ‘too fat’, while girls rated it higher. Both genders reported a higher self-esteem in the treatment-seeking sample than in the general population group with a perceived excess weight. The only dimension that was different between patients of both genders and other youth who felt ‘too fat’ was ‘friends’. The scores for the friends dimension were also significantly reduced in patients compared with the normal-weight youth, while compared with overweight youth the difference did not reach significance (p<0.10).

## Discussion

The main objective of our study was to compare self-reported and parent-reported HRQoL of treatment-seeking overweight, but not obese, children and adolescents with the HRQoL of age-matched youth in the general population, differentiated by objective and perceived weight status. We additionally explored whether results differed according to sociodemographic characteristics like age, gender, SES, and immigration.

### Treatment-seeking status

For conclusions about HRQoL differences due to treatment-seeking status patients should be compared with otherwise similar populations. In our overweight treatment-seeking sample, perceived weight was rated as ‘(much) too fat’ for nearly all participants. Compared with the group of overweight youth in the general population rated ‘too fat’, controlling for sociodemographic differences, treatment-seeking status in our sample was associated with impairments in only some HRQoL domains. Parents and adolescents differed in these perceived HRQoL limitations, and in some HRQoL domains patterns of results differed between treatment-seeking girls and boys.

Other studies that compared overweight/obese clinical samples with overweight/obese youth in the general population found more marked impairments in HRQoL than those observed here either using weight-specific HRQoL instruments [16,17] or, in extremely obese patients, using non-weight-specific HRQoL measures [18]. In another study using the generic PedsQL instrument, in contrast, HRQoL differences were not significant between a clinical sample and a community sample of obese children and adolescents after controlling for gender, age, and BMI z-score [14].

However, clinical samples were mainly compared with normal-weight controls. In our study differences between the treatment sample and the general population were generally higher for comparisons of patients with normal-weight youth than for comparisons between the treatment sample and overweight youth. Hughes et al. [39] found very similar results to our findings in obese treatment-seeking children compared with healthy controls with significant impairments in all HRQoL domains, while self-reported scores were less impaired and school and emotional well-being were the least affected. In another study on overweight and obese patients seeking outpatient training [40], self-reported HRQoL scores were only reduced in the ‘friends’ dimension compared with normal-weight students. On the other hand, a study with severely obese patients [41] found more pronounced HRQoL differences compared with normal-weight controls with large significant effects. Knöpfli et al. [42] also demonstrated significant impairments in all studied HRQoL dimensions in severely obese patients, but a direct comparison with a healthy sample was missing.

It may be inferred that treatment-seeking status in overweight or obese children and adolescents is associated with impaired HRQoL, but associations seem more manifest in obese than in overweight samples and may to a great extent be attributable to higher or extreme excess of weight in clinical samples. Furthermore, the perceived weight or body satisfaction of comparison groups was seldom taken into account.

### Perceived versus objective weight

Our results show that HRQoL differences between the patients and the general population were mainly due to differences between patients and other youth rated as ‘proper weight’. In other words, a large portion of HRQoL impairments were due to a perceived, but not objective, excess in weight.

We did not find any other study that compared a clinical sample with population groups differentiated according to perceived weight. However, our result that HRQoL impairments in overweight youth are mainly due to perceived instead of objective excess in weight supports the findings of Kurth and Ellert [22] regarding self-reported HRQoL of obese German youth. Correspondingly, a large U.S. study [23] found that the effects of obesity on psychological well-being were completely mediated by body dissatisfaction. Similar results were reported for female adolescents [25], where differences in psychosocial variables according to weight status lost significance after controlling for body satisfaction. In Dutch adolescents [24], the associations were found to be reversed, with obese adolescents reporting better psychological well-being than normal-weight counterparts after controlling for perceived weight.

### Domain-specific results

Previous studies found that physical well-being was usually significantly impaired in clinical samples, but most previous studies utilized obese samples. Our result that treatment-seeking status was only associated with lower physical well-being in boys, while girls had even higher scores than the general population, is a novel result that merits additional examination. These differences may especially concern moderately overweight youth who have less adverse physical impacts than individuals with greater excess weight who, therefore, have a higher risk of compromised physical well-being. It can be suspected that high physical well-being in overweight girls may encourage them to initiate life-style modifications that include regular exercising, whereas our results indicate that low physical well-being in boys may be an important constituent of the motivation to initiate weight loss treatment.

To our knowledge, our study is also the first to find higher self-reported self-esteem scores in treatment-seeking children and adolescents than in the general population. Although the decision to seek treatment in this age group presumably is considerably determined by the parents, and parent-perceived low self-esteem seems to be conducive to it, a child’s high self-esteem might encourage this decision. Another reason for higher self-esteem scores in treatment-seekers might be that more involved parenting fosters both children’s self-esteem and parents’ readiness to participate in a treatment program that requires commitment by parents. It was also supposed that overweight youth may hesitate to acknowledge negative impacts resulting from their weight [10].

However, self-proxy differences in self-esteem, which was generally overestimated by parents, were indeed *lower* in our clinical sample than in the general population. Parents of treatment-seeking youth may therefore perceive the self-esteem of their child more realistically than other parents. The awareness of a weight problem that leads to family talks and to the decision to seek treatment may also result in a greater awareness of a child’s self-esteem issues.

Among the social HRQoL domains, ‘friends’ was the domain that was most affected by treatment-seeking status in our sample, whereas familial well-being was less consistently reduced. Other studies reported significant impairments in the PedsQL social dimension in treatment-seekers [18,39,41,43] or in the overweight/obese in general [10,13,14], while studies that distinguished between impairments in the domains of family and friends mainly found impairments in the friends but not family domain in treatment-seeking youth [40].

Impairments in the friends dimension are likely due to negative stereotypes and stigmatization of overweight children and adolescents by peers [5]. Warschburger [7] suggested that, as a result of such peer stereotypes and stigmatization, obese children have fewer opportunities to develop social competence and supportive relationships, and thus, social well-being is negatively affected.

In summary, a universal pattern of impairments associated with treatment-seeking status in overweight youth cannot be deduced due to the limited number of studies available to date. However, impaired social well-being seems to be consistently affected in treatment-seekers independent of degree of overweight or source of information (self- vs. proxy-report).

### Self- versus proxy-reports

We found greater proxy-reported HRQoL impairments than self-reported impairments in this study, and this is in line with other studies on treatment-seeking youth [14,39,41]. Tsiros and colleagues [10] concluded that parents in general report lower HRQoL in overweight/obese adolescents than the adolescents themselves. Contrary to their result, we did not find proxy-reported scores to be consistently lower than self-reported scores in overweight or obese adolescents, but this was partly true for the treatment sample. This larger association of treatment-seeking status with parent-reported values can be explained by the vital role that parents play in health decision-making in this age group [40,44].

### Sociodemographic moderators

Concerning HRQoL associations with sociodemographic variables, our results are in accordance with those reported in the literature [10,37].

In general, associations between treatment-seeking status and weight were very similar for both genders and did not differ according to SES. For self-reported physical well-being, however, a significant interaction with gender was encountered. Thus, the decision to seek treatment for weight reduction appears to be partially motivated by different factors between boys and girls.

The effect of ethnic background on HRQoL has not been well studied. Other studies that considered ethnic background in comparisons with clinical samples reported conflicting results [18,39,45,46]. The number of immigrants in our sample was too small to generalize our finding of higher emotional well-being in treatment-seeking immigrants; however, because differential effects have been found in the past, it may be assumed that treatment-seeking status is associated with specific HRQoL patterns in ethnic minorities. Minority youth may have specific needs that might differ in particular ethnic groups, and should be further examined by future research.

### Strengths and limitations

Some strengths of our study should be highlighted. In contrast to previous studies, we focused on moderately overweight but not obese children and adolescents and included the perspectives of both adolescents and parents with regard to HRQoL. Because interventions for this target group are still missing, knowledge is limited. This group is particularly important because of the advantages in preventing rather than treating severe excess in weight. A further strength is the large representative sample that we were able to use for our comparison. To our knowledge, this is the first study to include not only objective but also subjective weight classifications in comparisons of a treatment sample with the general population. Finally, employing a multilevel approach to the data analysis, we allowed for clustering effects of the population sample and used all of the available data without having to exclude cases with single missing values.

However, some limitations should also be considered. First, the clinical sample was relatively small (especially for self-reports by adolescents) and consisted of participants of one treatment program in one area of Germany. Even if we controlled for deviations in sociodemographic characteristics, further unknown differences in the characteristics of the sample group may have influenced the results. The generalizability of our findings is therefore indeterminate, particularly in terms of whether our results can be generalized to treatment-seeking immigrants. Unequal group sizes are also a statistical concern, although inevitable when looking at naturally occurring groups in survey research. However, the employed hierarchical regression analysis approach allows for unequal group sizes. The reliability of some HRQoL subscales was unsatisfactory (especially ‘friends’), which may have reduced the accuracy of results. HRQoL scores were also not perfectly normally distributed. However, based on the computed robust standard errors and re-evaluation of results using transformed values, the distribution of scores should not have had considerable effects on our results. Finally, the measure of perceived weight status was rather crude. With a more sophisticated measure of perceived weight or body image, additional insights into HRQoL effects of subjective weight perceptions should be investigated in future studies.

### Practical implications

Our results have some practical implications for interventions addressing overweight children and adolescents.

In terms of treatment motivation for both parents and youth, social well-being (such as the anticipation of making friends in a training program or better acceptance by peers after losing weight) seems to play a crucial role. This motivation should therefore be adequately addressed by interventions.

Concerning gender differences in our results, especially in patients’ physical well-being, different treatment motives in girls and boys should be considered by gender-specific intervention content and marketing of interventions for overweight youth. Potentially varying needs of ethnic minority youth should also be taken into consideration when planning interventions, and this may involve preparatory exploration of targeted minority groups.

Because of the crucial influence parents may have on treatment decisions for their offspring, professionals planning or implementing treatments targeted at overweight children and adolescents should also take seriously the parent perceived well-being and problems of the child and, therefore, both perspectives should be assessed if possible [44]. Even if an intervention primarily aims at a better subjective well-being of the child, responding to issues that only parents perceive as problematic may be important to assure compliance with treatment and parental assistance in achieving life-style changes for the child.

The fact that perceived weight or body satisfaction seems to impact HRQoL more than objective weight status suggests that more attention should be paid to body self-perceptions in prevention and treatment programs for overweight in children and adolescents [23]. Health professionals should be aware of possible negative consequences for well-being, when informing overweight children and their families about excess weight of the child, while at the same time awareness of overweight may be required to facilitate treatment decisions. A dilemma arises as to how overweight youth can be engaged for interventions without further impairing their well-being [22]. On the one hand, it seems preferable to prevent further weight gain in moderately overweight youth by early treatment. On the other hand, in this target group impaired well-being due to perceived heavy weight actually may be the worst immediate consequence because manifest symptoms are rather unlikely in modestly overweight youngsters. It might be advantageous, therefore, to concentrate on counseling the parents first about overweight of the child and about possible negative effects of body dissatisfaction, since this may bring about treatment motivation without affecting the well-being of the child too much. Furthermore, programs for overweight children should include this topic and ensure that, besides weight reduction, self-acceptance and HRQoL are adequately addressed.

## Conclusions

HRQoL was impaired in treatment-seeking overweight children and adolescents according to self- as well as parent-reports and highlights certain limitations, especially in social well-being related to friends, as incentives to seek treatment.

However, differences between treatment-seekers and overweight youth likewise perceived as ‘too fat’ were small in contrast to differences when comparing treatment-seekers to normal-weight or overweight youth from the general population. The largest differences, in fact, were found in terms of perceived but not objective weight status or treatment status. Independent of objective weight, treatment status, and source of information (child vs. parent) girls and boys perceived as ‘too fat’ showed marked HRQoL impairments in all domains of well-being.

Future studies on this topic are required to validate the resulting patterns of HRQoL impairments with more diverse samples and more reliable measures. Moreover, further characteristics that may explain HRQoL impairments in treatment-seekers and those perceived as ‘too fat’ should be studied. Our findings may be useful for adapting treatments to suit the psychosocial needs of moderately overweight youth as well as to prompt the development of preventive action for youth who are dissatisfied with their weight.

## Endnote

^a^In an additional model we explored, if group effects might be explained by differences in physical activity, which was self-reported by adolescents of both samples. Although physical activity predicted HRQoL scores, including it did not change the magnitude of group effects. To reduce complexity and because there was no parent-reported information on physical activity, we did not include it in our analyses.

## Abbreviations

BMI: Body mass index (kg/m^2^); HRQoL: Health-related quality of life; KiGGS: German Health Interview and Examination Survey for Children and Adolescents; SES: Socioeconomic status.

## Competing interests

The authors declare that they have no competing interests.

## Authors’ contributions

EF participated in the acquisition of data for the Obeldicks light trial, conceptualized and performed the statistical analyses, and drafted the manuscript. PK and TR conceived the Obeldicks light study and its design and aided in the interpretation of the results. AS, KW, and TR participated in the acquisition of data. All authors critically revised the manuscript and read and approved the final manuscript.

## Pre-publication history

The pre-publication history for this paper can be accessed here:

http://www.biomedcentral.com/1471-2458/13/561/prepub

## Supplementary Material

Additional file 1 **Detailed interpretation of the hierarchical linear models from Tables** 3**and**4**.** Click here for file
