# Supplementary material for: Health-related quality of life in overweight German children and adolescents: do treatment-seeking youth have lower quality of life levels? Comparison of a clinical sample with the general population using a multilevel model approach
Source: BMC Public Health. 2013 Jun 8;13:561. doi: 10.1186/1471-2458-13-561 (PMC3683337; doi:10.1186/1471-2458-13-561)
Supplement: Additional file 1 — Detailed interpretation of the hierarchical linear models from Tables 3and4. [file 1471-2458-13-561-S1.pdf]

Additional file 1

Detailed interpretation of the hierarchical linear models from tables 3 and 4:

For the fixed portion of the model, the intercepts are the mean values and standard errors of the HRQoL subscales when all other values are “0”. The means, therefore, correspond to those of female participants in the Obeldicks light program (reference group) with average SES and age, as reported by their mothers. As expected, all HRQoL means were significantly different from “0”.

At level 2, each HRQoL subscale was predicted by gender (male vs. female), proxy (other vs. mother), immigration status (immigrant vs. non-immigrant), age (grand-mean centered, per year increase), SES score (grand-mean centered, per increase of 1; original scale ranging from 3 to 21), and group (compared with the Obeldicks light reference group). The coefficients correspond to the change in HRQoL with an increase of 1 in the corresponding predictor, with all other values held constant. A coefficient of 4.156 for gender in the parent model of physical well-being, for example, means that the predicted physical well-being score reported by mothers is 4.156 points higher in boys than girls, which is significant ($p<0.001$).

The multivariate hypothesis test examines whether all five dummy variables coding the group effect are simultaneously zero. It therefore tests the group effect as a whole, while the individual regression coefficients compare each particular group with the Obeldicks light reference group. There were no predictors at level 3.

For the random part of the model, level 2 variance components are the between-person standard deviations and variances of the six HRQoL subscales. Level 3 gives the standard deviations and variances of the between-sample-point variation in HRQoL scores that is separated from the between-subjects portion of the model.
